# Supplementary material for: Timing of dense granule biogenesis in asexual malaria parasites
Source: Microbiology (Reading). 2023 Aug 30;169(8):001389. doi: 10.1099/mic.0.001389 (PMC10482371; doi:10.1099/mic.0.001389)
Supplement: Supplementary material 1 [file mic-169-1389-s001.pdf]

Supplementary Table 1 Primers used in this study

Name	Sequence	Restriction site
CVO545	GTGAACAAATGAATTCAATAACATACAATTTCG	none
CV0550	GGAC**CTGCAG**AAAAAGTTAGTAAAGGAGAAGAAGATAATATGGCAAG	PstI
CVO551	GGAC**ACGCGT**TGCTTTATACAATTCATCCATTCCCATAACATCTGTAAATG	MluI
CVO576	GGAC**ACGCGT**GAAGGAAGAGGAAGTTTATTAACATGTGGAG	MluI
CVO577	GGAC**ACGCGT**TTAGAAGAACTCGTCAAGAAGGCG	MluI
CVO600	CAAAATGGTTAACAAAGAAGAAGCTCAGAG	none
TMR066A	CACATTTCTTTTTCATATATCTTGTTTTAAATATTTTATTTTCATAAAGAG	none

Supplementary Table 2 Proteins identified in malaria.tools search using RESA as query.

Gene ID	Protein
PF3D7_1115800	
PF3D7_0701900	
PF3D7_0935600	GIG
PF3D7_0220000	LSA3
PF3D7_1201200	Plasmodium RESA N-terminal
PF3D7_0702100	Plasmodium RESA N-terminal
PF3D7_1002100	PTP5
PF3D7_0501100	HSP40
PF3D7_1218500	DRPC
PF3D7_1016800	Plasmodium RESA N-terminal
PF3D7_0301800	
PF3D7_0605900	P GNS1/SUR4
PF3D7_0102700	MaTrA
PF3D7_0730800	
PF3D7_0814500	
PF3D7_1114200	Rab-GTPase-TBC domain.
PF3D7_0525100	SSF56801: AMP-binding enzyme.
PF3D7_1102800	ETRAMP11.2
PF3D7_1102700	ETRAMP11.1
PF3D7_0108500	ELM2 domain.
PF3D7_1010300	SDH4 0
PF3D7_0606000	
PF3D7_0702000	
PF3D7_0830500	TryThrA
PF3D7_1252600	SSF53474:Serine aminopeptidase, S33, esterase, putative
PF3D7_1105600	PTEX88
PF3D7_1343700	SSF117281: Kelch13
PF3D7_1237900	
PF3D7_0424500	FIKK4.1
PF3D7_1200700	AMP-binding enzyme. ACS7 acyl-CoA synthetase.
PF3D7_0902500	FIKK9.6
PF3D7_1149600	DnaJ domain, PF14308: X-domain of DnaJ-containing
PF3D7_1016700	Plasmodium RESA N-terminal
PF3D7_0423900	
PF3D7_0202200	PTP1
PF3D7_0805200	GAMER
PF3D7_1416500	PF00208: Glutamate/Leucine/Phenylalanine/Valine dehydrogenase
PF3D7_1001100	Plasmodium RESA N-terminal
PF3D7_0731100	GEXP20
PF3D7_0402100	SURF4.1
PF3D7_1479000	AMP-binding enzyme ACS1a
PF3D7_1433500	DNA gyrase B, TOP2
PF3D7_0202500	ETRAMP2
PF3D7_0219900	
PF3D7_1404800	
PF3D7_1478800	PF09715: Plasmodium protein of unknown function (Plasmod_dom_1)
PF3D7_0523000	MDR1
PF3D7_1404900	
PF3D7_1026600	
PF3D7_0314100	Vesicle transport v-SNARE protein N-terminus
PF3D7_1001900	PfJ23
PF3D7_1352900	
PF3D7_0702200	Pfam domain(s): PF12146: "Serine aminopeptidase, S33"
PF3D7_1226300	PF08282: haloacid dehalogenase-like hydrolase HAD2
PF3D7_1203700	PF00956: Nucleosome assembly protein (NAP) NAPL
PF3D7_1477800	PF00887: Acyl CoA binding protein. ACBP
PF3D7_0425100	hyp6
